# Early inspiris resilia valve failure in a patient with idiopathic pulmonary valve regurgitation

**DOI:** 10.1186/s13019-025-03398-7

**Published:** 2025-04-10

**Authors:** Benjamin D. Seadler, Hannah K. Holland, Jutta Novalija, Stefano Schena, G. Hossein Almassi

**Affiliations:** 1 Division of Cardiothoracic Surgery, Medical College of Wisconsin, 8701 Watertown Plank Road, Milwaukee, WI USA; 2 Department of Anesthesiology, Clement J. Zablocki Veterans Affairs Medical Center, Milwaukee, WI USA; 3 Division of Cardiothoracic Surgery, Clement J. Zablocki Veterans Affairs Medical Center, Milwaukee, WI USA

**Keywords:** Early prosthetic valve degeneration, Echocardiography, Pulmonary valve regurgitation, Pulmonary valve replacement

## Abstract

**Background:**

Pulmonary valve failure requiring replacement (PVR) is more commonly seen in children and young adults with congenital heart disease (CHD). Adults with CHD and pulmonary regurgitation have traditionally undergone PVR with bioprosthetic valves. The inspiris resilia bovine pericardial valve is an FDA-approved bioprosthesis for the aortic position with encouraging data on 7-year outcomes. Previous reports on PVR using the Inspiris valve in young patients with CHD have demonstrated early failure of the valve. We report the early failure of this device in an elder patient with idiopathic pulmonary regurgitation.

**Case presentation:**

The patient is a 69-year-old male with preoperative evaluation demonstrating idiopathic pulmonary valve regurgitation with moderately depressed right ventricular ejection fraction. The patient declined receiving a porcine valve, and therefore underwent PVR using the inspiris resilia (IR) valve due to known encouraging results when implanted in the aortic position in elder patients. A 27 mm IR valve was utilized, and intraoperative transesophageal echocardiography showed no regurgitation at the time of surgery. Surveillance echocardiography at 17 months, however, already demonstrated moderate to severe pulmonary prosthetic valve regurgitation.

**Conclusions:**

This report highlights an early failure of the IR valve used for PVR in an elder patient with idiopathic pulmonary regurgitation. Data on the IR valve in the pulmonic position is limited to mostly small cohorts of young patients with CHD, and immediate outcomes are nearly universally satisfactory. However, recent reports in this specific population indicate early recurrence of regurgitation in the IR cohort compared to patients managed with a commercially available porcine aortic bioprosthetic valve, when used in the pulmonic position. Our reported case suggests that utilization of IR in the pulmonic position should be approached with caution in elderly patients as well.

**Supplementary Information:**

The online version contains supplementary material available at 10.1186/s13019-025-03398-7.

## Background

Pulmonary valve insufficiency requiring surgical valve replacement (PVR) is more commonly seen in children and young adults with congenital heart disease (CHD). Idiopathic pulmonary regurgitation in the absence of infection, pulmonary hypertension or connective tissue disorders, is a rare condition. Adults with CHD and severe pulmonary regurgitation are traditionally managed by PVR with bioprosthetic valves [1]. The Resilia bovine pericardial valves (Edwards Lifesciences, Irvine, CA, USA) are FDA-approved for both aortic (Inspiris, IR) and mitral (Mitris) valve replacement and have been used among other models including the porcine bioprosthetic valves in pulmonary valve replacement due to lack of bioprostheses developed specifically for the pulmonary position. Compared to the previous iterations (Perimount and Magna) produced by the same manufacturer that have also been used off-label in PVR, the Resilia valve is a newer option postulated to offer enhanced anti-calcification technology that may potentially delay calcification and leaflet thickening, therefore positively impacting valve durability. IR is currently one of the most commonly implanted tissue valves, although in aortic position, with encouraging durability on 7-year follow-up data [2]. Recent reports on PVR using the IR valve, however, indicate early failure in young patients with CHD, with up to 48% of patients developing new prosthetic regurgitation at a mean follow-up of 16 months. Evidence on early and long-term outcomes in older adults without CHD is lacking. We report the early failure of an IR valve in the pulmonary position in an adult patient with idiopathic pulmonary regurgitation who underwent PVR 17 months earlier.

## Case presentation

The patient is a 69-year-old male, with a past medical history only remarkable for hypertension, who was assessed for a provisional diagnosis of idiopathic, severe pulmonary valve regurgitation. The initial diagnosis stems back to 2017, and he has been on yearly echocardiographic follow-up ever since. During this time interval, the pulmonary valve regurgitation had progressed from moderate to severe, with an elevated pulmonary artery systolic pressure (33 mmHg), normal left ventricular ejection fraction (LVEF, 54–60%), and a moderately enlarged right ventricular (RV) which had previously been normal in size. Cardiac MRI demonstrated a mildly enlarged right ventricular cavity with moderately depressed right ventricular ejection fraction (RVEF) of 36%. Despite the evidence on imaging tests, the patient denied fatigue, chest discomfort, or exertional dyspnea. Based on the above findings, the patient was referred for surgical evaluation. In discussing risks, benefits, and options related to a valve replacement procedure, the patient adamantly declined to use a porcine bioprosthetic valve. Plan was therefore made for PVR with an IR bovine pericardial valve. As part of preoperative work-up, coronary angiography did not demonstrate any coronary anatomic abnormalities or intraluminal disease.

**Fig. 1:**
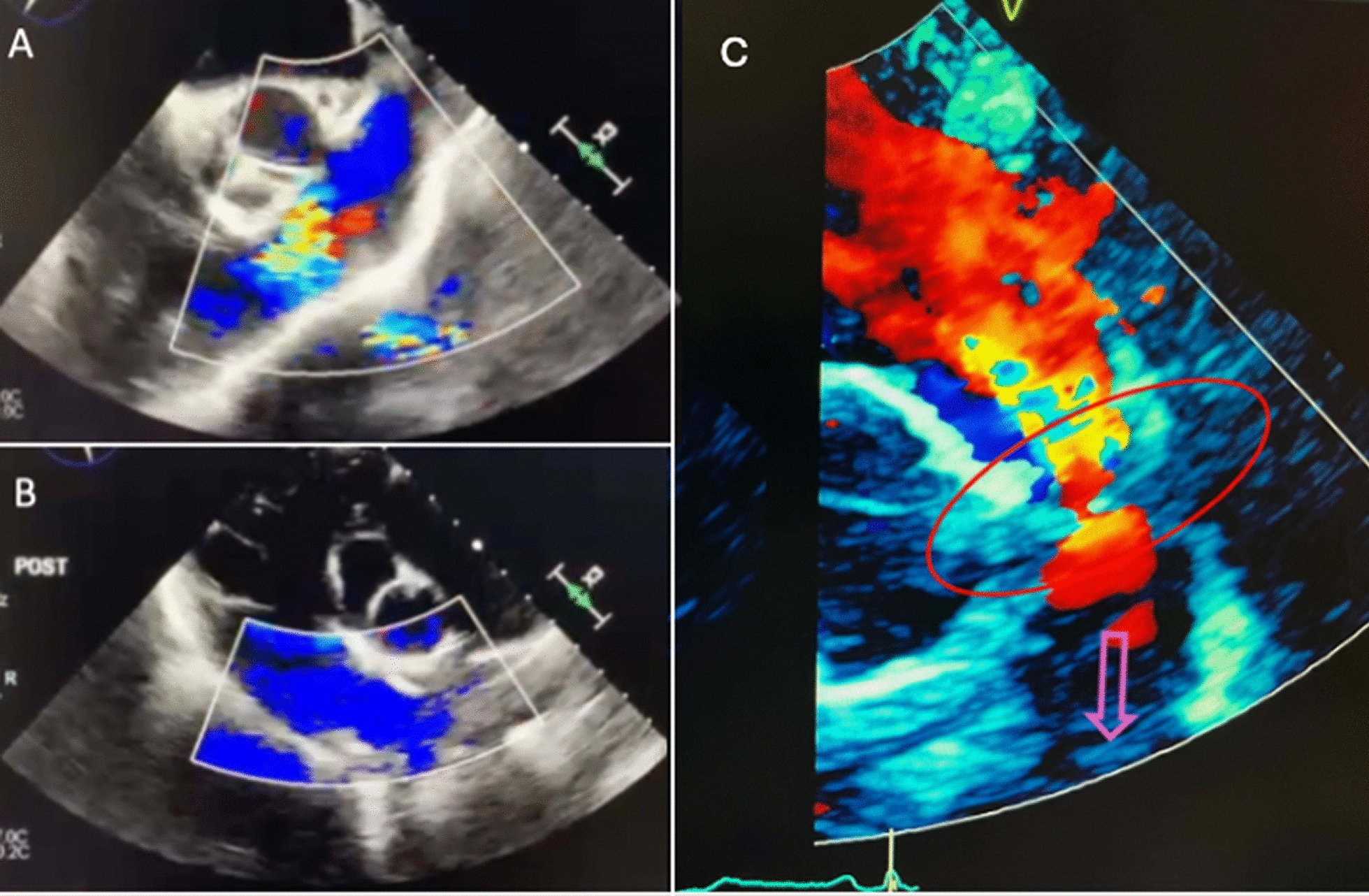
Intraoperative transesophageal echocardiogram of right ventricular inflow outflow view before **A** and after **B** pulmonary valve replacement showing severe pulmonary regurgitation before cardiopulmonary bypass and competent IR bioprosthetic valve after replacement. **C** Transthoracic echocardiography 17 months after implantation demonstrating moderate-severe regurgitation. Red oval: pulmonary valve; Purple arrow: pulmonary artery

**Fig. 2:**
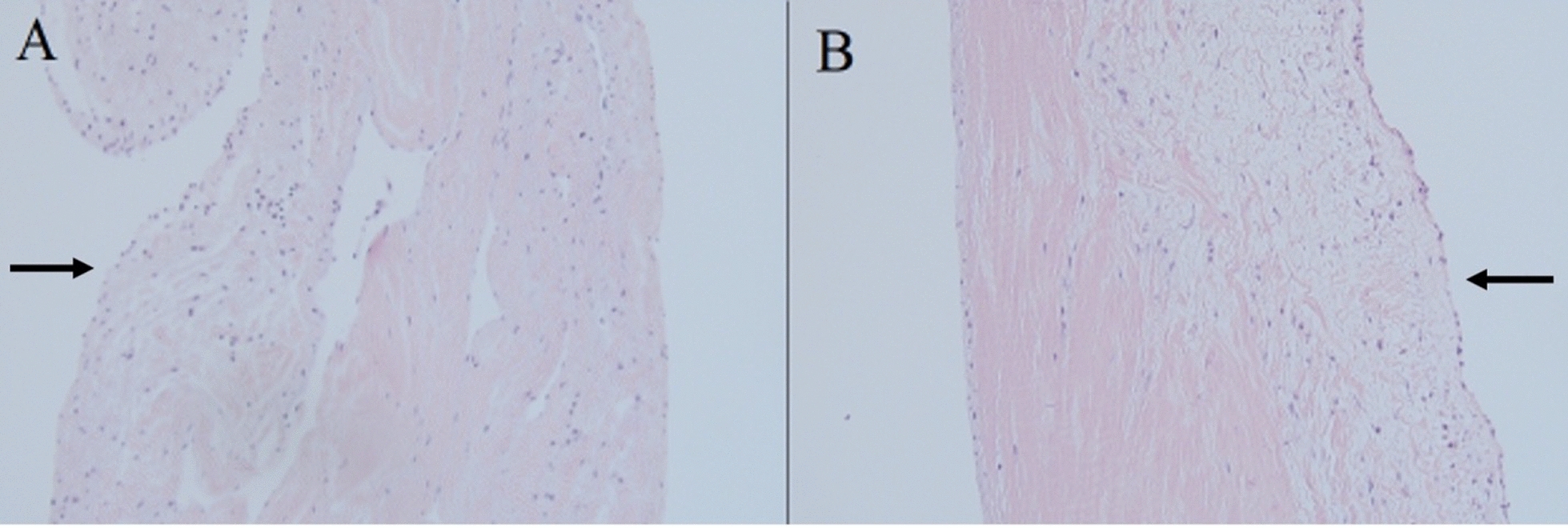
Histologic findings of the explanted valve demonstrating **A** myxoid degeneration and **B** chronic inflammation

Intraoperative transesophageal echocardiography (TEE) confirmed the presence of severe pulmonary regurgitation (Fig. 1A, Video 1). After cardioplegic arrest and longitudinal pulmonary arteriotomy, the pulmonary valve was carefully inspected and noted to be tri-leaflet with myxomatous and redundant leaflets but no fenestrations. Following resection of the pathologic leaflets, the valve annulus was sized and accommodated a 27 mm IR valve which was placed without difficulty followed by closure of the pulmonary artery with an on-lay bovine pericardial patch. Following separation from cardiopulmonary bypass, TEE revealed a well-seated bioprosthetic valve with no intra- or paravalvular regurgitation (Fig. 1B, Video 2). The patient tolerated the procedure well and was discharged home on postoperative day 5. He was subsequently maintained on a low-dose oral anticoagulation with Warfarin (goal INR 2–3) for 3 months, as well as lifelong 81-mg Aspirin. Final pathology of the specimen described the native pulmonary valve leaflets as characterized by myxoid degenerating stroma with minimal inflammation (Fig. 2). The patient, despite doing well from a clinical standpoint, was unable to return for follow-up imaging until 17 months after his initial surgery. Transthoracic echocardiography, at that time, revealed a mildly enlarged right ventricle with mildly reduced systolic function as well as moderate to severe pulmonary prosthetic valvular regurgitation with both an eccentric wall impinging jet and a central jet and no evidence of leaflet prolapse (Fig. 1C, Video 3). Subsequent cardiac MRI was performed, which revealed poor visualization of the IR prosthesis leaflets due to artifact. The patient was discussed at our complex structural heart conference and given the current lack of symptoms and patient preference, decision was taken to monitor the patient with serial evaluation for symptom development and echocardiographic follow-up.

## Discussion and conclusions

While there are no bioprosthetic valves specifically developed and approved for use in PVR, bioprostheses designed for aortic valve replacement are commonly used off-label in the pulmonary position [4]. Previously used bioprosthetic valves, including Sorin Mitroflow™ (Milan, Italy), Carpentier-Edwards Magna/Magna Ease™ and Carpentier-Edwards Perimount™ (Irvine, California) have demonstrated variable results in regard to pulmonary valve dysfunction and reintervention [1, 5]. The IR valve was approved by the FDA for use in the aortic position in 2017 and has similarly been used off-label in the pulmonary position in more recent years. In prior studies focusing specifically on IR valve use for PVR, nearly all initial IR implants were performed in children and young adults with CHD. They demonstrated encouraging results without paravalvular leaks or structural valve deterioration at the extent of the study period, which ranged from 1.6 to 2.7 years. [6, 7] Other investigations have reiterated these findings in the immediate postoperative period, but questioned the durability of the IR valve and indicated higher rates of early and long-term failure [3, 8]. Said et al. described a group of 27 patients with a history of CHD (mean age 22.3 years) necessitating pulmonic valve replacement. They found no prosthetic regurgitation on initial discharge in all patients, however, half of the cohort developed regurgitation over the 16-month follow-up period. The median duration from surgery to the discovery of regurgitation was 8 months, and 11% of patients underwent subsequent valve-in-valve transcatheter replacement [3]. Ragheb et al. performed a comparative retrospective analysis of midterm functional outcomes of the IR valve to an alternate commercially-available porcine valve, the Mosaic (Medtronic Inc, Minneapolis, MN) placed in pulmonary position in a larger cohort of 225 patients (mean age 15.7 years) with CHD and pulmonary regurgitation. In the study, sixty-two patients underwent replacement with the IR valve and 163 with the Mosaic valve. The study again revealed excellent short-term echocardiographic outcomes at hospital discharge. However, it demonstrated more clinically significant pulmonary regurgitation at mid-term follow-up (within 4 years of implantation) in the IR group compared to the Mosaic group. Patients that developed moderate or severe PVR at 2 and 3 years, respectively, were 12% and 31% in the IR cohort, but 5% and 7% in the Mosaic cohort. [8]

More recent matched analysis by Nguyen et al. in a cohort of 227 patients (median age 19 years), again all with CHD, were followed to a median time of 26.6 months. In this analysis, 120 patients received the IR valve, and 107 received a non-IR valve. Propensity score matching was performed and 2-year freedom from valve failure was 54% in the IR group vs 79% in the non-IR group (*P* = 0.03). When assessing IR outcomes, 18-month failure was higher when implanted into the native right ventricular outflow tract (41%) vs as part of a synthetic conduit (14%, *P* = 0.03^)^. [9] A review by Patel et al. of the Emory University institutional data on CHD patients receiving PVR in 10 years from the Society of Thoracic Surgeons database comparing porcine and bovine pericardial valves at mid-term follow-up demonstrated a higher 5-year cumulative incidence of valve dysfunction in the bovine pericardial cohort (39% vs 17%). The median age for the pericardial group at valve implantation was 22.0 (IQR 14–33) years vs. 16.0 (IQR 11–23) years (*P* < 0.001) in the porcine cohort. This analysis did not include IR valves specifically but did include bovine pericardial prostheses from the same manufacturer as the IR. [10]

The cause of these premature failures continues to be debated. It has been postulated that the over-sizing of such bioprosthetic valves, potentially in an attempt to allow for future valve-in-valve procedures, may distort functional anatomy and predispose to regurgitation [11]. Such approach indeed finds no justification given the fact that IR valves that are 25 mm or smaller in diameter are built with an expandable frame to allow for subsequent transcatheter valve replacement with even larger diameter valves. Reiterating concerns about anatomic distortion leading to regurgitation, some groups report less regurgitation by using a posterior-superior angulation during implantation to lie in a more natural anatomic position with the pulmonary outflow tract [11, 12]. Of note, none of the patients that had PVR within a synthetic conduit had early regurgitation in the cohort previously described by Said et al., and failure rates by Nguyen et al. were significantly lower when implanted in a conduit vs the native RVOT [3, 9]. The impact of age at the time of implantation and on structural valve degeneration is debated but has not been fully elucidated.^12,13^ While multiple theories and potential preventative steps exist, there is no consensus as to the exact cause of early regurgitation reported in multiple large single-center studies in patient with CHD.

The case presented is notable given the scarcity of available evidence of early IR prosthetic valve failure in an older adult without CHD. While most existing literature focused on younger populations undergoing PVR secondary to CHD, this patient’s pulmonary regurgitation was presumed to be idiopathic with no structural heart defects identified on pre-operative evaluation. The valve was implanted prior to availability of studies indicating early IR prosthetic valve failure, and this report further adds to this literature given this different patient profile. With the current published reports on the shorter durability the IR valve in the pulmonary position, such use should be approached with caution.

## Supplementary Information

Supplementary file 1: (Video 1: Intraoperative transesophageal echocardiogram prior to pulmonary valve replacement demonstrating severe pulmonary regurgitation and moderate right ventricular enlargement.)

Supplementary file 2: (Video 2: Intraoperative transesophageal echocardiogram after pulmonary valve replacement displays a well-seated valve with no regurgitation or peri-valvular leak.)

Supplementary file 3: (Video 3: Transthoracic echocardiogram 17 months after bioprosthetic pulmonary valve replacement demonstrating moderate to severe regurgitation with an eccentric wall impinging jet and central jet.)

## Data Availability

No datasets were generated or analysed during the current study.

## Abbreviations

CHD: Congenital heart disease
INR: International normalized ratio
IR: Inspiris resilia
LVEF: Left ventricular ejection fraction
PVR: Pulmonary valve replacement
RV: Right ventricle
RVOT: Right ventricular outflow tract
TEE: Transesophageal echocardiography
